# Genetic Variations in the Serotoninergic System Contribute to Body-Mass Index in Chinese Adolescents

**DOI:** 10.1371/journal.pone.0058717

**Published:** 2013-03-15

**Authors:** Chunhui Chen, Wen Chen, Chuansheng Chen, Robert Moyzis, Qinghua He, Xuemei Lei, Jin Li, Yunxin Wang, Bin Liu, Daiming Xiu, Bi Zhu, Qi Dong

**Affiliations:** 1 State Key Laboratory of Cognitive Neuroscience and Learning, Beijing Normal University, Beijing, China; 2 Department of Psychology and Social Behavior, University of California Irvine, Irvine, California, United States of America; 3 Department of Biological Chemistry and Institute of Genomics and Bioinformatics, University of California Irvine, Irvine, California, United States of America; 4 Department of Psychology, University of Southern California, Los Angeles, California, United States of America; The University of Texas M. D. Anderson Cancer Center, United States of America

## Abstract

**Objective:**

Obesity has become a worldwide health problem in the past decades. Human and animal studies have implicated serotonin in appetite regulation, and behavior genetic studies have shown that body mass index (BMI) has a strong genetic component. However, the roles of genes related to the serotoninergic (5-hydroxytryptamine,5-HT) system in obesity/BMI are not well understood, especially in Chinese subjects.

**Subjects and Design:**

With a sample of 478 healthy Chinese volunteers, this study investigated the relation between BMI and genetic variations of the serotoninergic system as characterized by 136 representative polymorphisms. We used a system-level approach to identify SNPs associated with BMI, then estimated their overall contribution to BMI by multiple regression and verified it by permutation.

**Results:**

We identified 12 SNPs that made statistically significant contributions to BMI. After controlling for gender and age, four of these SNPs accounted for 7.7% additional variance of BMI. Permutation analysis showed that the probability of obtaining these findings by chance was low (p = 0.015, permuted for 1000 times).

**Conclusion:**

These results showed that genetic variations in the serotoninergic system made a moderate contribution to individual differences in BMI among a healthy Chinese sample, suggesting that a similar approach can be used to study obesity.

## Introduction

A decade ago, the World Health Organization warned about a growing obesity epidemic and listed more than 30 diseases that are causally related to obesity [1]. Globally, approximately 1.6 billion adults are either overweight (BMI [weight in kilogram divided by the square of height in meter]≥25) or obese (BMI ≥30) [2]. In fact, the rates of obesity have tripled in developing countries in the past 20 years [3]. Moreover, childhood obesity is also increasing rapidly worldwide [4].

Although many environmental factors (e.g. freely available high-calorie food, sedentary life style, low socio-economic status and high-danger neighborhood environment) predispose individuals to gaining weight [5], [6], [7], [8], genetic factors also contribute to energy homeostasis or appetite, which can lead to obesity. Family, twin, and adoption studies indicate that 24%–90% of human BMI variation is due to genetic factors [9], [10], [11], [12], [13], [14]. Recent molecular genetic studies have identified many genes that regulate appetite or energy balance (e.g, *FTO, MC4R, SH2B1*, and serotonin related genes ) and have robust associations with obesity or BMI [15], [16].

Because serotonin can regulate appetite by activating pro-opiomelanocortin (POMC) neurons, which play a key role in the regulation of feeding by sending anorectic signals to the periventricular nucleus (PVN) and other brain areas associated with energy homeostasis [17], serotonin as well as related genes are often tested for association with weight gain and obesity. Indeed, a strong negative correlation between blood 5-HT concentration and body mass was found both in mice [18] and in human [19]. Studies of *SERT* knockout mice have uncovered *SERT* as a candidate gene for obesity, with *SERT* mutant (SCL6A4−/−) mice becoming obese [20]. This polymorphism has also been associated in some studies with eating disorder [21], [22], [23] and obesity [24], [25], although other studies showed no association between the *5-HTTLPR* polymorphism and weight regulation [26], [27], [28]. In terms of the 5-HT receptor genes, the serotonin (5-HT) receptor *HTR2C* was demonstrated to play a role in modulating appetite behavior using knock-out mice [29], [30], normal population [31] and patients [32], [33], [34], [35], although some studies [36], [37] failed to replicate that result. *HTR1B* [38], [39], *HTR2A* [40], [41], *HTR3B* [42] were also reported to be associated with body mass or obesity. *MAOA* was also found to influence body mass [43] or obesity [44].

Although these serotonin-related genes have been identified as being relevant to body mass and obesity, the results have not always been consistent and the size of their effects has been typically small, far less than previously estimated 24%–90% heritability. There may be many reasons for these inconsistent results and small effect sizes. One most likely reason is polygenicity. Complex quantitative traits are influenced by many genes, each with a small effect. As early as 1918, Fisher proposed this polygenic model that combined many genes of small effects to yield the continuous variation for most quantitative traits [45]. Recently, some studies have successfully applied the polygenic model by combining effects of the whole genome [46], [47], [48] or effects of genes within a pathway [49], [50], [51]. Since several serotonin-related genes exert their effect on BMI, it is likely that their effects are cumulative. The current study used a system-level approach to examine the role of the serotoninergic system in BMI/obesity.

Another possible reason for inconsistent results may be the heterogeneity in samples across studies. Subjects in different studies differ in their health status, age, sex, and ethnicity, which might have confounded the relations between genes and BMI. For example, associations between *5HTR2A* and BMI are found in obese [40] and anorexia nervosa patients [41] but not in healthy controls. Similarly, *5HTTLPR* was associated with BMI in non-elderly (<65 yr) stroke patients but not in elderly patients (> or = 65 yr). An association was observed between *MAOA* and obesity among white and Hispanic American subjects, but not among African–American subjects [44]. Thus it is important to control for these potential confounding factors.

The current study adopted the system-level approach to examine the role of the serotoninergic system in body mass in a relatively homogenous sample (in terms of age, health status, and ethnicity). We enrolled a sample of young healthy Han Chinese subjects, genotyped polymorphisms within the serotonin system, and calculated their BMI. Specifically, we selected 136 polymorphic loci (including 134 SNPs and 2 VNTR polymorphisms) to cover a substantial portion (by LD) of the common variations within known genes of the 5-HT system to estimate the additive and multiplicative contributions of these genes on BMI.

## Materials and Methods

### Participants

Four hundred and eighty healthy Chinese college students (mean age = 20, SD = 1) were recruited from Beijing Normal University, Beijing, China. They had normal or corrected-to-normal vision, and had no history of neurological or psychiatric problems according to self-report. None of them were identified as having alcohol or nicotine dependence according to the Alcohol Use Disorders Identification Test [52] and the Fagerström Test for Nicotine Dependence [53]. Two participants were excluded because of poor genotyping results. A written consent form was obtained from each subject after a full explanation of the study procedure. This study was approved by the IRB of the State Key Laboratory of Cognitive Neuroscience and Learning at Beijing Normal University, China.

### BMI Measurements

Height and weight of subjects were self-reported. BMI was calculated as weight (kg) divided by the square of height (m). Self-reported data on weight and height have been used by previous large-scale studies on body mass and proved to be highly reliable in calculating BMI [22], [38], [46], [54], [55], [56], [57]. Furthermore, all students including all of our participants were given an annual physical examination at the beginning of the academic year in September and they were informed of their height and weight. Self-report data on height and weight were collected in December.

### Genetic Analysis

#### Gene selection

We selected 25 genes and 136 associated polymorphisms (134 SNPs and 2 VNTR polymorphisms) distributed across the synthesis, degradation, transporter, and receptor subsystems of the 5-HT system. 5-HT synthesis involves converting the tryptophan (via TPH) to 5-hydroxytryptophan (5-HTP), followed by subsequent hydroxylation (by TPH) to 5-HT. We included two genes related to 5-HT synthesis: tryptophan hydroxylase (*TPH1* and *TPH2*, with three SNPs each). For the degradation subsystem, released 5-HT is directly broken down at the synapse into inactive metabolites by two enzymes, COMT and MAO (including MAOA and MAOB). We included catechol-O-methyl transferase gene (*COMT*, with 7 SNPs) and monoamine oxidase genes (*MAOA*, with 5 SNPs and 1 VNTR, and *MAOB* with 3 SNPs). The 5-HT transporter includes (1) SLC6A4, an integral membrane-spanning protein that pumps the neurotransmitter serotonin from synaptic spaces into presynaptic neurons and (2) VMAT, a transport protein integrated into the membrane of intracellular vesicles of presynaptic neurons, which acts to transport monoamines into the synaptic vesicles. We included *SLC6A4* (7 SNPs plus 5HTTLPR), *VMAT1* (SLC18A1, 9 SNPs), and *VMAT2* (SLC18A2, 5 SNPs). For the receptor subsystem, we included all 17 genes (with the respective number of SNPs in parentheses): *HTR1A* (2), *HTR1B* (2), *HTR1D* (13), *HTR1F* (5), *HTR2A* (21), *HTR2B* (6), *HTR2C* (3), *HTR3A* (1), *HTR3B* (2), *HTR3C* (3), *HTR3D* (4), *HTR3E* (2), *HTR4* (10), *HTR5A* (4), *HTR5B* (2), *HTR6* (5), and *HTR7* (7). Together, the above 25 genes represent all major genes involved in these four 5-HT subsystems in humans [58]. Details about these genes and the selected loci can be found in Table S1.

#### Genotyping techniques

The SNPs were genotyped using the standard Illumina Golden Gate Genotyping protocol (see Illumina Golden-Gate Assay Protocol for details, http://www.southgene.com.cn; Shanghai South Gene Technology Co., Ltd, Shanghai, China). In addition, three genetic markers (*5HTTLPR, MAOA VNTR*, and *COMT* rs4680) were ascertained by standard PCR procedures [59], [60], [61].

#### Gene data preprocessing

Two subjects with more than 10% null genotyping were excluded. In addition to automatic calling of genotypes, Illumina genotyping platform supplied a quantitative quality measure known as the GenCall score. It measures how close a genotype is to the center of the cluster of other samples assigned to the same genotypes, compared with the centers of the clusters of the other genotypes. This measure ranges from 0 to 1, with a higher score indicating a more reliable result. The conventional cutoff point is.25 [62]. Of the 63574 genotypes (133 SNPs by 478 subjects) in the current study, 120 genotypes (0.2%) were excluded because their GenCall scores were lower than.25.

Additional data cleaning included the treatment of low-frequency alleles. For SNPs with either heterozygote or minor homozygote found in fewer than 10 (about 2%) participants, these two genotype groups were combined. If the combined group still had fewer than 10 participants, the SNP(s) were excluded from further analysis. SNPs that showed no polymorphisms were also deleted. In order to examine sample representativeness, Hardy-Weinberg equilibrium (HWE) index was calculated using the Chi square test and setting *df* to 1. Since males have only one X chromosome, only females were included in HWE calculation for SNPs located on X chromosome. Five of the SNPs showed significant HW disequilibrium (p<0.01). The inclusion of both tSNPs and additional SNPs in regions detected in selection screens [63], [64] resulted in high LD among a number of SNPs. Thirty-one SNPs included in initial analysis were excluded from multiple regression analysis because of their high LD with other adjacent SNPs (R^2^>0.8, calculated with Plink [65]), yielding a final list of 105 polymorphisms for the main data analyses. It is worth mentioning that the “redundant” SNPs showed the same or almost the same results as the linked SNPs, confirming the association. Table S1 shows the details about all 136 polymorphic loci (134 SNPs and 2 VNTRs) included in our study: location (rs number, chromosome, position), gene, serotonin subsystem, allele polymorphism and frequency, Hardy Weinberg equilibrium, linkage disequilibrium and deleted SNPs. Finally, genetic relatedness of subjects was checked following Anderson et.al. [66] protocol using Plink. We used all 240 unrelated autosome SNPs (r^2^<0.8) available in the larger project of these subjects and set the threshold of 0.95 (personal communication with Drs. Anderson and Zondervan). We found no pair of subjects showing high relatedness (all PI_HAT smaller than or equal to 0.5).

### Data Analysis

The goal of the current study was to understand the relation between individual differences in BMI and genetic variations in the 5-HT system in healthy subjects. Moving beyond the single-gene or a small number of haplotypes approaches used in typical molecular behavior genetics research, this study used the system-level approach [50] to examine the overall contributions of the serotoninergic system (characterized by the major genes and their associated loci) on BMI.

Briefly, the analysis includes three steps: First, ANOVA was used to screen polymorphism loci that showed nominal significance (*p<0.05*) on BMI; these loci were then entered into a regression model to estimate their overall contribution to BMI after controlling for gender and age; and lastly the regression model was verified by permutation. In this study, we built two kinds of regression models. In model 1 (main effects), we included the loci with significant main effects based on the ANOVA results and used the forward stepwise method to build the model. Gender and age were entered as control variables. To run multiple regression analyses, all SNPs were coded in a linear way, i.e. the major homozygote, heterozygote, and minor homozygote were coded into 1, 2, and 3, respectively (SNPs on X chromosome were coded as 1 and 3 for major and minor allele, and 3 for female heterozygotes). In addition, the MAOA VNTR was coded as 1 for the 3 repeat and 3 for the 4 repeat in males and 1 for 3 repeat homozygotes and 3 for others in females. In model 2, all two-way interactions of these loci in model 1 were added using forward stepwise method. Permutation was done 1000 times by shuffling BMI (along with gender and age) across subjects, and the probability of getting a larger R^2^ in the shuffled data than in the real data was defined as *p* value of the model.

## Results

The mean BMI for our sample was 20.5 kg/m^2^ (SD = 2.4), ranging from 16.3 to 37.5. According to WHO BMI classification, there were 93 (71 female) underweight participants (BMI <18.5), 359 (192 female) normal weight participants (18.5≤ BMI <25), and 26 (8 female) overweight participants (BMI ≥25). The BMI distribution in the present study was comparable to other studies with Chinese college students [67], [68]. Males (21.14±2.44) had significantly higher BMI than females (20.00±2.67; *t*(476) = 5.30, *p* = 1.8E−7), which was consistent with previous findings in healthy young Chinese [69].

Of the 105 SNPs, 12 showed significant main effects with uncorrected *p*<0.05. Specifically, individuals with the following genotypes showed lower BMI than those with alternative alleles: homozygous for the major allele of rs13166761 (HTR4), rs1018079 (SLC18A1(VMAT1)), rs11214769 (HTR3B), rs977003 (HTR2A), rs2224721 (HTR2A), rs2192371 (HTR2C), rs4911871 (HTR2C), or rs2270638 (VMAT1); or heterozygous/minor allele homozygous for rs6651806 (MAOB), rs5905512 (MAOB); or homozygous for the minor allele of rs7904569 (HTR7) or rs6644065 (HTR2C) (see Table 1, and Table S2 for effects of all loci). These SNPs were used in a regression analysis to build model 1 (main effects). There was no significant gender-by-SNP interaction except rs5905512 (see Table 1), and this SNP did not contribute to regression model 1, so we included gender, but not gender-by-gene interactions, as a covariate in the following analysis.

**Table 1:** Means and standard deviations of BMI for each polymorphism, and main effects and post hoc comparisons of SNPs that showed significant main effects and were used in subsequent multiple regression analysis.

SNP	Subsystem	Gene	Maj	Mean	SD	n	Het	Mean	SD	n	Min	Mean	SD	n	F	p	mh	mm	hm^a^	Gene bygenderinteractionF(p)
rs6651806	Degradation	MAOB	AA	20.63	2.45	381	AC	19.95	2.16	97	CC				6.32	0.01	b			0.40 (0.53)
rs5905512		MAOB	AA	20.76	2.62	284	AG	20.10	2.01	194	GG				9.02	<0.01	b			0.02 (0.89)
rs1018079	Transport	SLC18A1	AA	20.29	2.26	303	AG	20.72	2.32	156	GG	22.10	4.35	18	5.92	<0.01	0.07	<0.01	0.02	0.34 (0.71)
rs2270638		SLC18A1	AA	20.33	2.47	344	AG	20.92	2.20	133	GG				5.77	0.02	b			0.27 (0.60)
rs977003	Receptor	HTR2A	AA	20.29	2.30	299	AC	20.92	2.62	151	CC	20.40	2.19	28	3.45	0.03	0.01	0.82	0.29	0.14 (0.87)
rs2224721		HTR2A	CC	20.12	2.12	216	AC	20.79	2.61	209	AA	20.87	2.51	53	4.85	0.01	<0.01	0.04	0.82	0.13 (0.88)
rs2192371		HTR2C	AA	20.61	2.42	242	AG	19.79	1.80	124	GG	21.03	2.78	112	8.62	<0.01	<0.01	0.12	<0.01	0.95 (0.33)
rs6644065		HTR2C	AA	20.45	2.26	373	AG	20.05	2.81	68	GG	21.74	2.77	37	6.30	<0.01	0.21	<0.01	<0.01	6.06 (0.01)
rs4911871		HTR2C	AA	20.39	2.23	350	AG	20.17	2.74	76	GG	21.68	2.76	51	7.44	<0.01	0.47	<0.01	<0.01	1.09 (0.30)
rs11214769		HTR3B	AA	20.31	2.22	335	AG	20.88	2.85	124	GG	21.21	2.20	19	3.46	0.03	0.02	0.11	0.58	1.65 (0.19)
rs13166761		HTR4	GG	20.38	2.23	244	AG	20.79	2.64	202	AA	19.48	1.81	32	4.74	0.01	0.07	0.05	<0.01	0.54 (0.59)
rs7904569		HTR7	AA	20.50	2.38	206	AG	20.69	2.53	215	GG	19.72	1.85	57	3.64	0.03	0.43	0.03	0.01	1.34 (0.26)

Note: Empty cells mean no such genotypes were found in our sample. Maj: Major allele; Het: Heterozygote; Min: Minor allele.

a Results (*p* values) of post hoc comparisons. mh = Maj versus Het, mm = Maj versus Min, hm = Het versus Min.

b Post hoc comparison was not run because there were only 2 groups for this locus.

Table 2 shows the results of the multiple regression analysis. On the first step, two control variables (gender and age) were entered. Together they accounted for 5.6% variance of BMI. On the second step, forward stepwise regression resulted in four of the 12 SNPs to be included in the regression equation, showing that they made unique contributions to explaining variance in BMI. Together these SNPs accounted for 7.7% additional variance, yielding a total R^2^ of.13, *F*(6,455) = 11.61, *p* = 4.08E−12.

**Table 2:** Regression models.

Regressor	Gene	Beta	T	P
Gender		−0.24	−5.39	0.00
Age		0.02	0.47	0.64
rs1018079	SLC18A1(VMAT1)	0.16	3.63	0.00
rs11214769	HTR3B	0.14	3.08	0.00
rs2224721	HTR2A	0.12	2.76	0.01
rs4911871	HTR2C	0.12	2.81	0.01

Note: ‘Gene’ is the corresponding gene for each SNP; ‘beta’ is the standardized regression coefficient, ‘T’ and ‘P’ are t-test results.

**Figure 1:**
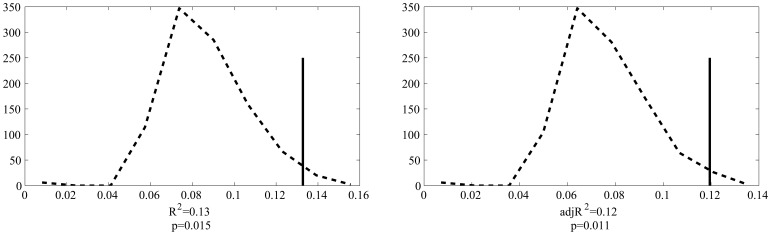
Permutation results for R^2^ (left panel) and adjusted R^2^ (right panel). Dashed line represents distribution of R2 obtained from randomized data and solid line represents the observed R^2^.

Permutation results are shown in Figure 1. Based on 1000 permutations, the probability of attaining the R^2^ or adjusted R^2^ found in our model was 0.015 and 0.011, respectively.

We then added potential interactive effects to investigate whether additional variance in BMI can be accounted for by gene–gene interactions. In this analysis, we first entered the control variables (gender and age) and the four SNPs in model 1 and finally their two-way interactions using the stepwise procedure. For the four SNPs that entered model 1, there were 6 potential interactions. None of the interaction terms made significant and unique contributions to the model.

## Discussion

Based on the system-level analysis of 5-HT neurotransmitter genes, we identified 12 SNPs of the 5-HT-related genes showing nominal effects on BMI. Four of these SNPs made significant unique contributions to BMI even after controlling for gender and age. This result has two significant implications. First, the current study revealed a significant role for genes in the 5-HT system on BMI among Chinese, confirming that body mass is likely to be influenced to some extent by the serotoninergic system. Second, our results supported the idea that BMI may be determined by many loci. Only by summing up their overall effects can we understand the genetic basis of a complex trait such as body mass. This approach can estimate the overall contribution of genes within a pathway and can help to explain the missing heritability [46].

We found that 12 SNPs of seven genes (*MAOB*, *SLC18A1*(*VMAT1*), *HTR2A*, *HTR2C*, *HTR3B*, *HTR4*, and *HTR7*) were significantly associated with BMI. As summarized in the introduction, previous studies have already found evidence, although not always consistent, of association between the *HTR2A* and *HTR2C* genes and BMI. However, other genes we identified have not been tested previously in BMI-related studies to the best of our knowledge.

*VMAT1* is expressed primarily in neuroendocrine cells such as the adrenal medulla and pineal gland [70], [71], [72]. As early as in 1999, Hayashi et al. [73] found that VMAT1 was responsible for the storage of 5-hydroxytryptamine in rat pinealocytes. Mammalian pinealocytes contain more 5-HT than any other cells. Upon stimulation by norepinephrine (NE), the internal 5-HT is released and then stimulates serotonin N-acetyltransferase activity via the 5-HT2 receptor, resulting in increased melatonin output [73]. Melatonin has been found to be involved in energy metabolism and body weight control in both animals [74], [75] and humans [76]. Decreased activity of the melanocortin system produces a marked orexigenic effect, while increased activity increases α-melanocyte-stimulating hormone (α-MSH) release leading to satiation and a termination of feeding. On the other hand, the missence variation Thr136Ile in the *VMAT1/SLC18A1* gene was found to be associated with anxiety-related personality traits [77] and anxiety has been shown to be associated with obesity [78], [79] or BMI [80]. Previous studies have found that VMAT plays an important role in the life cycle of ghrelin and obestatin in the A-like cells of the stomach [81], [82], and ghrelin and obestatin have effects on food intake and energy balance. Therefore, we speculate that the *VMAT1* gene may have an effect on BMI through melatonin output, ghrelin, obestatin or anxiety mood. This gene accounted for the largest proportion of the variance of BMI in our study (Table 2).

The 5-HT3 receptor has been suggested to be involved in anxiety, depression, pain, alcohol dependence, and eating disorders [42], [83]. The *HTR3B* gene encodes the B-subunit of the type 3 serotonin receptor (5-HT3), a ligand-gated ion channel that is known to be involved in gut motility and peristalsis. Thus the *HTR3B* gene may regulate BMI because gut motility is associated with numerous gastrointestinally derived peptides with significant effects on food intake and energy balance [84]. Many studies have also reported that the *5-HT3B* polymorphism is associated with the incidence of major depression [85], efficiency of the antidepressant treatment [86], and the incidence and severity of nausea after paroxetine treatment of psychiatric patients [87]. Although the specific biological mechanisms are not well understood, our results indicate that *HTR3B* gene polymorphism may influence body mass via gut motility or mood.

Our analysis also showed that *HTR2C* and *HTR2A* are possible factors influencing BMI in Chinese subjects, as have been reported by previous studies. Different from the most often studied C759T polymorphism associated with weight gain [31], [32], [33], [34], [35], [88], three SNP we found related to BMI are all located in the intron region of *HTR2C*. First, there is strong evidence for an interaction between leptin and the 5-HTergic system [88]. Second, McCarthy et al. showed a strong effect of *HTR2C* polymorphism −759G>A on circulating leptin levels after adjusting for body fat. Other studies also suggested that serotonin influences food intake because of variations in the HTR2C receptor [89], [90]. Similarly, previous researchers have also found an association between a polymorphism −1438G4A (rs6311) in the regulatory region of the *HTR2A* gene and alteration in food intake [91], [92], [93], [94], but the significant SNP rs2224721 we found is intronic. Recent studies suggest that polymorphic variation in the *HTR2A* gene may be associated with abdominal obesity and the metabolic syndrome, and that *HTR2A* may be linked to the stability of the stress-related system (i.e., the serotonin-hypothalamic-pituitary-adrenal system) [95], [96].

Several limitations of the current study need to be mentioned. First, height and weight of the subjects were self-reported. Although other large-scale studies also used self-reported data [22], [38], [46], [54], [55], [56], [57] and previous research showed high correlations (*r* = .92) between BMI calculated from self-reports and that from actual measurements [56], it would still be better to measure weight and height during the experiment. Second, this study focused only on healthy Han Chinese college students, so these results may or may not be generalized to other populations (e.g., clinical samples, other ethnic groups). Third, the sample size of the current study is modest. As power calculations based on the effect sizes of established variants have suggested that increasing the sample size would likely lead to the discovery of additional variants [97], follow-up research needs to expand the sample size and validate the results. Fourth, we examined only the serotonin system and accounted for only 7% of the variance of BMI, there is much more “missing heritability” of BMI (estimated 24–90%) to be accounted for. Other genetic systems [15], [16] and behavioral factors (e.g, such as physical activity, sedentary life-style, and dietary patterns) as well as their interactions need to be examined. Furthermore, gene-environment interaction studies are needed to understand epigenetic factors in BMI.

In conclusion, we used a system-level approach to identify several genetic SNPs associated with variations in BMI. This analysis provides further evidence for the association between genetic variants in the serotonin pathway and BMI. Because current lifestyle interventions are largely ineffective in addressing the challenges of growing obesity [98], [99], new insights into the biology of obesity are critically needed to guide the development and application of future therapies and interventions.

## Supporting Information

Table S1 **Detailed information of the loci used in this study.**
(DOC)

Table S2 **Means and standard deviations of BMI for each polymorphism, and main effects and post hoc comparisons of each locus.**
(DOC)
