# Supplementary material for: Genetic Variations in the Serotoninergic System Contribute to Body-Mass Index in Chinese Adolescents
Source: PLoS One. 2013 Mar 15;8(3):e58717. doi: 10.1371/journal.pone.0058717 (PMC3598805; doi:10.1371/journal.pone.0058717)
Supplement: Table S1 — Detailed information of the loci used in this study. (DOC) [file pone.0058717.s001.doc]

**Table S1. Detailed information of the loci used in this study**

| SNP | Chr | Position | Gene | Subsystem | Maj | N | Het | N | Min | N | Miss | HWE p | LD  r^2^>0.8 | Deleted |
| --- | --- | --- | --- | --- | --- | --- | --- | --- | --- | --- | --- | --- | --- | --- |
| rs1800532 | 11 | 18004392 | TPH1 | Synthesis | CC | 138 | AC | 230 | AA | 110 | 0 | 0.45 |  |  |
| rs10488683 | 11 | 18010121 | TPH1 |  | AA | 168 | AG | 208 | GG | 102 | 0 | 0.01 |  |  |
| rs11024449 | 11 | 18014461 | TPH1 |  | GG | 455 | AG | 23 | AA | 0 | 0 | 0.59 |  |  |
| rs11179000 | 12 | 70624895 | TPH2 |  | AA | 349 | AT | 0 | TT | 129 | 0 | 0.00 |  |  |
| rs7955501 | 12 | 70636293 | TPH2 |  | AA | 173 | AT | 225 | TT | 80 | 0 | 0.64 |  |  |
| rs1487275 | 12 | 70696559 | TPH2 |  | AA | 191 | AC | 223 | CC | 64 | 0 | 0.93 |  |  |
| rs737866 | 22 | 18310109 | COMT | Degradation | AA | 246 | AG | 197 | GG | 35 | 0 | 0.60 |  |  |
| rs5993883 | 22 | 18317638 | COMT |  | AA | 172 | AC | 234 | CC | 72 | 0 | 0.60 |  |  |
| rs740603 | 22 | 18325177 | COMT |  | AA | 153 | AG | 240 | GG | 71 | 14 | 0.14 |  |  |
| rs2239393 | 22 | 18330428 | COMT |  | AA | 198 | AG | 218 | GG | 62 | 0 | 0.87 |  |  |
| COMT3 | 22 | 18331270 | COMT |  | VV | 256 | VM | 178 | MM | 30 | 14 | 0.90 |  |  |
| rs4646316 | 22 | 18332132 | COMT |  | GG | 209 | AG | 210 | AA | 58 | 1 | 0.64 | 120 | Y |
| rs165774 | 22 | 18332561 | COMT |  | GG | 369 | AG | 103 | AA | 6 | 0 | 0.69 |  |  |
| rs929095 | 23 | 43247894 | MAOA |  | GG | 196 | CG | 128 | CC | 154 | 0 | 0.48 |  |  |
| rs1181286 | 23 | 43300472 | MAOA |  | CC | 396 | AC | 53 | AA | 29 | 0 | 0.52 |  |  |
| rs1181289 | 23 | 43325208 | MAOA |  | TT | 396 | AT | 52 | AA | 30 | 0 | 0.95 | 125 | Y |
| rs5906974 | 23 | 43436265 | MAOA |  | GG | 217 | AG | 140 | AA | 121 | 0 | 0.13 |  | Y |
| rs909525 | 23 | 43438146 | MAOA |  | GG | 217 | AG | 140 | AA | 120 | 1 | 0.13 | 127 |  |
| MAOA_VNTR | 23 |  | MAOA |  | VV | 210 | VM | 130 | MM | 129 | 9 | 0.61 |  |  |
| rs1799836 | 23 | 43512943 | MAOB |  | AA | 352 | AG | 77 | GG | 49 | 0 | 0.22 |  |  |
| rs6651806 | 23 | 43573908 | MAOB |  | AA | 381 | AC | 68 | CC | 29 | 0 | 0.71 |  |  |
| rs5905512 | 23 | 43611338 | MAOB |  | AA | 284 | AG | 117 | GG | 77 | 0 | 0.17 |  |  |
| rs1042173 | 17 | 25549137 | SLC6A4 | Transport | CC | 306 | AC | 146 | AA | 26 | 0 | 0.13 |  |  |
| rs4325622 | 17 | 25550601 | SLC6A4 |  | GG | 306 | AG | 146 | AA | 26 | 0 | 0.13 | 110 | Y |
| rs3794808 | 17 | 25555919 | SLC6A4 |  | AA | 309 | AG | 142 | GG | 27 | 0 | 0.05 | 110 111 | Y |
| rs140701 | 17 | 25562658 | SLC6A4 |  | AA | 308 | AG | 142 | GG | 28 | 0 | 0.04 | 110 111 112 | Y |
| rs4583306 | 17 | 25562841 | SLC6A4 |  | GG | 310 | AG | 141 | AA | 27 | 0 | 0.05 | 110 111 112 113 | Y |
| rs2020942 | 17 | 25571040 | SLC6A4 |  | GG | 402 | AG | 73 | AA | 3 | 0 | 0.87 |  |  |
| rs8076005 | 17 | 25571336 | SLC6A4 |  | AA | 355 | AG | 111 | GG | 12 | 0 | 0.35 |  |  |
| 5HTTLPR |  |  | 5HTT |  | MM | 246 | VM | 187 | VV | 42 | 3 | 0.45 |  |  |
| rs1497020 | 8 | 20046706 | SLC18A1(VMAT1) |  | AA | 133 | AG | 235 | GG | 110 | 0 | 0.75 |  |  |
| rs1018079 | 8 | 20049580 | SLC18A1(VMAT1) |  | AA | 303 | AG | 156 | GG | 18 | 1 | 0.71 |  |  |
| rs12545707 | 8 | 20049620 | SLC18A1(VMAT1) |  | GG | 458 | AG | 20 | AA | 0 | 0 | 0.64 |  |  |
| rs903997 | 8 | 20049996 | SLC18A1(VMAT1) |  | GG | 142 | CG | 235 | CC | 101 | 0 | 0.84 |  |  |
| rs2270650 | 8 | 20052292 | SLC18A1(VMAT1) |  | GG | 319 | AG | 140 | AA | 19 | 0 | 0.46 | 60 | Y |
| rs3779671 | 8 | 20067165 | SLC18A1(VMAT1) |  | GG | 219 | CG | 189 | CC | 70 | 0 | 0.01 |  |  |
| rs13258461 | 8 | 20073883 | SLC18A1(VMAT1) |  | GG | 169 | AG | 215 | AA | 94 | 0 | 0.09 |  |  |
| rs952858 | 8 | 20076882 | SLC18A1(VMAT1) |  | GG | 434 | CG | 3 | CC | 41 | 0 | 0.00 |  |  |
| rs2270638 | 8 | 20081297 | SLC18A1(VMAT1) |  | AA | 344 | AG | 128 | GG | 5 | 1 | 0.07 |  |  |
| rs363338 | 10 | 118999379 | SLC18A2(VMAT2) |  | GG | 310 | AG | 154 | AA | 14 | 0 | 0.32 |  |  |
| rs363222 | 10 | 119009438 | SLC18A2(VMAT2) |  | CC | 212 | CG | 210 | GG | 54 | 2 | 0.85 |  |  |
| rs4752045 | 10 | 119009680 | SLC18A2(VMAT2) |  | GG | 148 | CG | 281 | CC | 49 | 0 | 0.00 |  |  |
| rs363225 | 10 | 119014492 | SLC18A2(VMAT2) |  | GG | 147 | AG | 241 | AA | 89 | 1 | 0.58 |  |  |
| rs363226 | 10 | 119015202 | SLC18A2(VMAT2) |  | CC | 393 | CG | 78 | GG | 7 | 0 | 0.18 |  |  |
| rs878567 | 5 | 63291747 | HTR1A | Receptor | GG | 316 | AG | 152 | AA | 10 | 0 | 0.09 |  |  |
| rs10042486 | 5 | 63297085 | HTR1A |  | AA | 316 | AG | 152 | GG | 10 | 0 | 0.09 | 41 | Y |
| rs6297 | 6 | 78228660 | HTR1B |  | AA | 387 | AG | 85 | GG | 6 | 0 | 0.59 |  |  |
| rs6298 | 6 | 78229711 | HTR1B |  | AA | 131 | AG | 229 | GG | 118 | 0 | 0.37 |  |  |
| rs2776822 | 1 | 23365369 | HTR1D |  | GG | 158 | AG | 242 | AA | 78 | 0 | 0.36 |  | Y |
| rs2746553 | 1 | 23365833 | HTR1D |  | AA | 158 | AG | 242 | GG | 78 | 0 | 0.36 | 6 | Y |
| rs2776815 | 1 | 23383171 | HTR1D |  | AA | 268 | AG | 178 | GG | 32 | 0 | 0.74 |  | Y |
| rs6704440 | 1 | 23385634 | HTR1D |  | CC | 334 | AC | 131 | AA | 13 | 0 | 0.97 |  |  |
| rs641032 | 1 | 23386567 | HTR1D |  | AA | 268 | AG | 178 | GG | 32 | 0 | 0.74 | 8 | Y |
| rs604030 | 1 | 23390814 | HTR1D |  | AA | 268 | AG | 178 | GG | 32 | 0 | 0.74 | 8 10 | Y |
| rs676643 | 1 | 23393927 | HTR1D |  | GG | 273 | AG | 173 | AA | 32 | 0 | 0.52 | 8 10 11 | Y |
| rs674386 | 1 | 23394422 | HTR1D |  | GG | 269 | AG | 177 | AA | 32 | 0 | 0.69 | 8 10 11 12 |  |
| rs2746561 | 1 | 23399139 | HTR1D |  | AA | 273 | AG | 173 | GG | 32 | 0 | 0.52 | 8 10 11 12 13 | Y |
| rs598438 | 1 | 23404150 | HTR1D |  | GG | 165 | AG | 241 | AA | 72 | 0 | 0.29 | 6 7 | Y |
| rs1738475 | 1 | 23409478 | HTR1D |  | GG | 159 | CG | 242 | CC | 77 | 0 | 0.34 | 6 7 15 |  |
| rs627304 | 1 | 23410142 | HTR1D |  | AA | 159 | AG | 242 | GG | 77 | 0 | 0.34 | 6 7 15 16 | Y |
| rs2903545 | 1 | 23413695 | HTR1D |  | AA | 159 | AG | 242 | GG | 77 | 0 | 0.34 | 6 7 15 16 17 | Y |
| rs7630716 | 3 | 88116161 | HTR1F |  | CC | 250 | CG | 190 | GG | 38 | 0 | 0.82 |  | Y |
| rs9863076 | 3 | 88116629 | HTR1F |  | CC | 250 | AC | 190 | AA | 38 | 0 | 0.82 | 27 | Y |
| rs1503433 | 3 | 88119890 | HTR1F |  | AA | 250 | AC | 190 | CC | 38 | 0 | 0.82 | 27 28 | Y |
| rs2016224 | 3 | 88124340 | HTR1F |  | AA | 250 | AG | 190 | GG | 38 | 0 | 0.82 | 27 28 29 | Y |
| rs7652406 | 3 | 88135928 | HTR1F |  | GG | 251 | AG | 189 | AA | 38 | 0 | 0.77 | 27 28 29 30 |  |
| rs7997012 | 13 | 46309986 | HTR2A |  | GG | 265 | AG | 173 | AA | 40 | 0 | 0.13 |  |  |
| rs977003 | 13 | 46313002 | HTR2A |  | AA | 299 | AC | 151 | CC | 28 | 0 | 0.13 |  |  |
| rs6561332 | 13 | 46317821 | HTR2A |  | CC | 412 | AC | 63 | AA | 3 | 0 | 0.73 |  |  |
| rs6561333 | 13 | 46318313 | HTR2A |  | GG | 150 | AG | 230 | AA | 98 | 0 | 0.57 |  |  |
| rs9567739 | 13 | 46322945 | HTR2A |  | CC | 143 | CG | 218 | GG | 117 | 0 | 0.06 |  |  |
| rs655888 | 13 | 46326182 | HTR2A |  | GG | 150 | AG | 214 | AA | 113 | 1 | 0.03 | 93 | Y |
| rs7984966 | 13 | 46327447 | HTR2A |  | AA | 435 | AG | 43 | GG | 0 | 0 | 0.30 |  |  |
| rs1885884 | 13 | 46328277 | HTR2A |  | GG | 266 | CG | 177 | CC | 35 | 0 | 0.46 |  |  |
| rs9526240 | 13 | 46329472 | HTR2A |  | GG | 457 | AG | 21 | AA | 0 | 0 | 0.62 |  | Y |
| rs2224721 | 13 | 46330155 | HTR2A |  | CC | 216 | AC | 209 | AA | 53 | 0 | 0.82 |  |  |
| rs6561335 | 13 | 46331498 | HTR2A |  | AA | 459 | AG | 19 | GG | 0 | 0 | 0.66 | 97 |  |
| rs1928042 | 13 | 46335217 | HTR2A |  | AA | 429 | AC | 47 | CC | 2 | 0 | 0.56 |  |  |
| rs2770293 | 13 | 46336975 | HTR2A |  | GG | 295 | AG | 160 | AA | 23 | 0 | 0.83 |  | Y |
| rs9534501 | 13 | 46337977 | HTR2A |  | GG | 303 | AG | 153 | AA | 22 | 0 | 0.63 | 101 |  |
| rs2770296 | 13 | 46338561 | HTR2A |  | AA | 265 | AG | 181 | GG | 32 | 0 | 0.88 |  |  |
| rs985933 | 13 | 46353864 | HTR2A |  | GG | 202 | AG | 209 | AA | 66 | 1 | 0.31 |  |  |
| rs927544 | 13 | 46354052 | HTR2A |  | AA | 236 | AG | 192 | GG | 50 | 0 | 0.24 | 104 | Y |
| rs1328684 | 13 | 46364231 | HTR2A |  | AA | 406 | AG | 68 | GG | 4 | 0 | 0.54 |  |  |
| rs2296973 | 13 | 46364782 | HTR2A |  | CC | 212 | AC | 208 | AA | 58 | 0 | 0.53 |  |  |
| rs2070040 | 13 | 46365627 | HTR2A |  | GG | 205 | AG | 210 | AA | 62 | 1 | 0.48 | 107 | Y |
| rs953451 | 15 | 36027756 | HTR2A |  | CC | 237 | CG | 209 | GG | 32 | 0 | 0.12 |  |  |
| rs17619600 | 2 | 231684704 | HTR2B |  | AA | 299 | AG | 165 | GG | 14 | 0 | 0.12 |  |  |
| rs6437000 | 2 | 231685771 | HTR2B |  | AA | 166 | AC | 238 | CC | 74 | 0 | 0.46 |  | Y |
| rs10194776 | 2 | 231688263 | HTR2B |  | AA | 174 | AG | 233 | GG | 71 | 0 | 0.62 | 22 | Y |
| rs16827801 | 2 | 231689021 | HTR2B |  | AA | 120 | AG | 243 | GG | 115 | 0 | 0.71 |  |  |
| rs1549339 | 2 | 231691070 | HTR2B |  | AA | 170 | AG | 235 | GG | 73 | 0 | 0.58 | 22 23 |  |
| rs17586428 | 2 | 231697099 | HTR2B |  | AA | 288 | AG | 173 | GG | 17 | 0 | 0.14 |  |  |
| rs2192371 | 23 | 113796095 | HTR2C |  | AA | 242 | AG | 124 | GG | 112 | 0 | 0.94 |  |  |
| rs6644065 | 23 | 113808442 | HTR2C |  | AA | 373 | AG | 68 | GG | 37 | 0 | 0.50 |  |  |
| rs4911871 | 23 | 113903396 | HTR2C |  | AA | 350 | AG | 76 | GG | 51 | 1 | 0.93 |  |  |
| rs2276302 | 11 | 113355350 | HTR3A |  | AA | 374 | AG | 97 | GG | 6 | 1 | 0.92 |  |  |
| rs11214769 | 11 | 113295878 | HTR3B |  | AA | 335 | AG | 124 | GG | 19 | 0 | 0.09 |  |  |
| rs1176746 | 11 | 113307811 | HTR3B |  | GG | 206 | AG | 225 | AA | 46 | 1 | 0.17 |  |  |
| rs6808122 | 3 | 185255515 | HTR3C |  | AA | 274 | AG | 179 | GG | 25 | 0 | 0.54 |  |  |
| rs6766410 | 3 | 185257456 | HTR3C |  | AA | 177 | AC | 240 | CC | 61 | 0 | 0.14 |  |  |
| rs6807362 | 3 | 185260704 | HTR3C |  | GG | 241 | CG | 204 | CC | 33 | 0 | 0.25 |  |  |
| rs939334 | 3 | 185232425 | HTR3D |  | AA | 247 | AG | 188 | GG | 42 | 1 | 0.47 |  |  |
| rs10937159 | 3 | 185234995 | HTR3D |  | AA | 240 | AC | 183 | CC | 55 | 0 | 0.03 |  |  |
| rs6792482 | 3 | 185236723 | HTR3D |  | GG | 126 | AG | 236 | AA | 116 | 0 | 0.79 |  |  |
| rs1467257 | 3 | 185238225 | HTR3D |  | CC | 290 | AC | 149 | AA | 27 | 12 | 0.18 |  |  |
| rs7627615 | 3 | 185301110 | HTR3E |  | AA | 276 | AG | 177 | GG | 25 | 0 | 0.62 |  |  |
| rs7432211 | 3 | 185301849 | HTR3E |  | AA | 187 | AG | 235 | GG | 56 | 0 | 0.17 |  |  |
| rs7733401 | 5 | 147813474 | HTR4 |  | CC | 123 | AC | 263 | AA | 92 | 0 | 0.02 |  |  |
| rs3995090 | 5 | 147826008 | HTR4 |  | CC | 270 | AC | 174 | AA | 34 | 0 | 0.41 |  |  |
| rs4597955 | 5 | 147827466 | HTR4 |  | AA | 432 | AG | 44 | GG | 2 | 0 | 0.45 |  |  |
| rs10051356 | 5 | 147845252 | HTR4 |  | GG | 176 | CG | 232 | CC | 70 | 0 | 0.65 |  |  |
| rs1883074 | 5 | 147853943 | HTR4 |  | GG | 145 | AG | 245 | AA | 88 | 0 | 0.38 |  |  |
| rs13166761 | 5 | 147927481 | HTR4 |  | GG | 244 | AG | 202 | AA | 32 | 0 | 0.25 |  |  |
| rs6873382 | 5 | 147938456 | HTR4 |  | AA | 151 | AG | 247 | GG | 80 | 0 | 0.21 |  |  |
| rs7711800 | 5 | 147963048 | HTR4 |  | GG | 128 | AG | 241 | AA | 109 | 0 | 0.83 |  |  |
| rs6580561 | 5 | 147990262 | HTR4 |  | AA | 234 | AG | 197 | GG | 47 | 0 | 0.56 |  |  |
| rs1972644 | 5 | 147999741 | HTR4 |  | AA | 165 | AG | 220 | GG | 90 | 3 | 0.28 |  |  |
| rs1800883 | 7 | 154493524 | HTR5A |  | CC | 133 | CG | 219 | GG | 125 | 1 | 0.08 |  |  |
| rs6320 | 7 | 154493554 | HTR5A |  | AA | 183 | AT | 225 | TT | 70 | 0 | 0.95 |  |  |
| rs732050 | 7 | 154504328 | HTR5A |  | AA | 302 | AG | 157 | GG | 19 | 0 | 0.80 |  |  |
| rs1440449 | 7 | 154506190 | HTR5A |  | AA | 191 | AC | 219 | CC | 68 | 0 | 0.68 |  |  |
| rs11676829 | 2 | 118371773 | HTR5B |  | AA | 361 | AG | 107 | GG | 10 | 0 | 0.53 |  |  |
| rs2245686 | 2 | 118371917 | HTR5B |  | GG | 276 | AG | 165 | AA | 36 | 1 | 0.11 |  |  |
| rs10917509 | 1 | 19864653 | HTR6 |  | AA | 309 | AG | 2 | GG | 167 | 0 | 0.00 |  |  |
| rs4912138 | 1 | 19866072 | HTR6 |  | AA | 138 | AG | 216 | GG | 124 | 0 | 0.04 |  |  |
| rs6658108 | 1 | 19868078 | HTR6 |  | GG | 280 | AG | 168 | AA | 30 | 0 | 0.48 |  |  |
| rs3790757 | 1 | 19870495 | HTR6 |  | GG | 348 | AG | 121 | AA | 9 | 0 | 0.68 |  |  |
| rs9659997 | 1 | 19875105 | HTR6 |  | GG | 302 | AG | 157 | AA | 19 | 0 | 0.80 | 1 | Y |
| rs7904569 | 10 | 15650570 | HTR7 |  | AA | 206 | AG | 215 | GG | 57 | 0 | 0.94 |  |  |
| rs4933194 | 10 | 92501347 | HTR7 |  | GG | 192 | AG | 227 | AA | 59 | 0 | 0.52 |  |  |
| rs11596518 | 10 | 92544991 | HTR7 |  | AA | 304 | AG | 154 | GG | 20 | 0 | 0.93 |  |  |
| rs1573935 | 10 | 92554807 | HTR7 |  | AA | 132 | AG | 241 | GG | 105 | 0 | 0.80 |  |  |
| rs10881838 | 10 | 92580213 | HTR7 |  | AA | 164 | AG | 230 | GG | 84 | 0 | 0.83 |  |  |
| rs12249377 | 10 | 92582936 | HTR7 |  | CC | 344 | AC | 124 | AA | 10 | 0 | 0.76 |  |  |
| rs10785973 | 10 | 92588151 | HTR7 |  | CC | 240 | AC | 201 | AA | 37 | 0 | 0.57 |  |  |

Note: Chr = Chromosome, Maj= major homozygote, N= number of subjects, Het=heterozygote, Min=minor homozygote, Miss = number of subjects missing genotype

^ξ^ SNPs with the same numbers are clusters with high LD (r^2^>0.8), “Y” in “deleted” column means this SNP was deleted because of high LD
