# Supplementary material for: Genetic Variations in the Serotoninergic System Contribute to Body-Mass Index in Chinese Adolescents
Source: PLoS One. 2013 Mar 15;8(3):e58717. doi: 10.1371/journal.pone.0058717 (PMC3598805; doi:10.1371/journal.pone.0058717)
Supplement: Table S2 — Means and standard deviations of BMI for each polymorphism, and main effects and post hoc comparisons of each locus. (DOC) [file pone.0058717.s002.doc]

**Table S2**. Means and standard deviations of BMI for each polymorphism, and main effects and post hoc comparisons of each locus.

| SNP | Subsystem | Gene | Maj | Mean | SD | n | Het | Mean | SD | n | Min | Mean | SD | n | F | p | mh | mm | hm^a^ |
| --- | --- | --- | --- | --- | --- | --- | --- | --- | --- | --- | --- | --- | --- | --- | --- | --- | --- | --- | --- |
| rs1800532 | Synthesis | TPH1 | CC | 20.51 | 2.20 | 138 | AC | 20.48 | 2.30 | 230 | AA | 20.50 | 2.87 | 110 | 0.01 | 0.99 | 0.90 | 0.98 | 0.93 |
| rs10488683 |  | TPH1 | AA | 20.55 | 2.70 | 168 | AG | 20.44 | 2.28 | 208 | GG | 20.51 | 2.16 | 102 | 0.09 | 0.91 | 0.67 | 0.90 | 0.82 |
| rs11024449 |  | TPH1 | GG | 20.46 | 2.42 | 455 | AG | 21.10 | 2.12 | 23 | AA |  |  |  | 1.52 | 0.22 | 0.22 |  |  |
| rs11179000 |  | TPH2 | AA | 20.56 | 2.48 | 349 | AT | 20.31 | 2.20 | 129 | TT |  |  |  | 1.05 | 0.31 | 0.31 |  |  |
| rs7955501 |  | TPH2 | AA | 20.45 | 2.08 | 173 | AT | 20.45 | 2.38 | 225 | TT | 20.69 | 3.09 | 80 | 0.33 | 0.72 | 0.99 | 0.46 | 0.45 |
| rs1487275 |  | TPH2 | AA | 20.50 | 2.35 | 191 | AC | 20.47 | 2.27 | 223 | CC | 20.55 | 3.04 | 64 | 0.03 | 0.97 | 0.93 | 0.87 | 0.82 |
| rs737866 | Degradation | COMT | AA | 20.48 | 2.34 | 246 | AG | 20.53 | 2.23 | 197 | GG | 20.37 | 3.67 | 35 | 0.07 | 0.93 | 0.83 | 0.80 | 0.72 |
| rs5993883 |  | COMT | AA | 20.51 | 2.35 | 172 | AC | 20.60 | 2.29 | 234 | CC | 20.13 | 2.91 | 72 | 1.05 | 0.35 | 0.71 | 0.26 | 0.15 |
| rs740603 |  | COMT | AA | 20.56 | 2.38 | 153 | AG | 20.51 | 2.24 | 240 | GG | 20.38 | 3.01 | 71 | 0.14 | 0.87 | 0.83 | 0.60 | 0.70 |
| rs2239393 |  | COMT | AA | 20.54 | 2.37 | 198 | AG | 20.53 | 2.21 | 218 | GG | 20.21 | 3.13 | 62 | 0.48 | 0.62 | 0.98 | 0.36 | 0.36 |
| COMT3 |  | COMT | VV | 20.53 | 2.51 | 256 | VM | 20.33 | 2.25 | 178 | MM | 20.47 | 2.13 | 30 | 0.39 | 0.68 | 0.38 | 0.89 | 0.76 |
| rs4646316 |  | COMT | GG | 20.51 | 2.38 | 209 | AG | 20.51 | 2.21 | 210 | AA | 20.35 | 3.15 | 58 | 0.12 | 0.89 | 0.98 | 0.64 | 0.66 |
| rs165774 |  | COMT | GG | 20.50 | 2.38 | 369 | AG | 20.48 | 2.53 | 109 | AA |  |  |  | < 0.01 | 0.96 | b |  |  |
| rs929095 |  | MAOA | GG | 20.75 | 2.67 | 196 | CG | 20.32 | 2.20 | 282 | CC |  |  |  | 3.78 | 0.05 | b |  |  |
| rs1181286 |  | MAOA | CC | 20.54 | 2.29 | 396 | AC | 20.29 | 2.93 | 82 | AA |  |  |  | 0.70 | 0.40 | b |  |  |
| rs1181289 |  | MAOA | TT | 20.54 | 2.29 | 396 | AT | 20.29 | 2.93 | 82 | AA |  |  |  | 0.70 | 0.40 | b |  |  |
| rs5906974 |  | MAOA | GG | 20.61 | 2.30 | 217 | AG | 20.39 | 2.50 | 261 | AA |  |  |  | 0.97 | 0.33 | b |  |  |
| rs909525 |  | MAOA | GG | 20.61 | 2.30 | 217 | AG | 20.38 | 2.49 | 260 | AA |  |  |  | 1.11 | 0.29 | b |  |  |
| MAOA_VNTR |  | MAOA | VV | 20.67 | 2.26 | 210 | VM | 20.38 | 2.55 | 259 | MM |  |  |  | 1.66 | 0.20 | b |  |  |
| rs1799836 |  | MAOB | AA | 20.62 | 2.57 | 352 | AG | 20.14 | 1.85 | 126 | GG |  |  |  | 3.79 | 0.05 | b |  |  |
| rs6651806 |  | MAOB | AA | 20.63 | 2.45 | 381 | AC | 19.95 | 2.16 | 97 | CC |  |  |  | 6.32 | 0.01 | b |  |  |
| rs5905512 |  | MAOB | AA | 20.76 | 2.62 | 284 | AG | 20.10 | 2.01 | 194 | GG |  |  |  | 9.02 | < 0.01 | b |  |  |
| rs1042173 | Transport | SLC6A4 | CC | 20.39 | 2.13 | 306 | AC | 20.69 | 2.92 | 146 | AA | 20.58 | 2.39 | 26 | 0.74 | 0.48 | 0.23 | 0.71 | 0.83 |
| rs4325622 |  | SLC6A4 | GG | 20.39 | 2.13 | 306 | AG | 20.69 | 2.92 | 146 | AA | 20.58 | 2.39 | 26 | 0.74 | 0.48 | 0.23 | 0.71 | 0.83 |
| rs3794808 |  | SLC6A4 | AA | 20.41 | 2.14 | 309 | AG | 20.68 | 2.93 | 142 | GG | 20.42 | 2.40 | 27 | 0.63 | 0.53 | 0.27 | 0.98 | 0.61 |
| rs140701 |  | SLC6A4 | AA | 20.43 | 2.16 | 308 | AG | 20.65 | 2.90 | 142 | GG | 20.43 | 2.35 | 28 | 0.42 | 0.66 | 0.37 | 1.00 | 0.66 |
| rs4583306 |  | SLC6A4 | GG | 20.42 | 2.15 | 310 | AG | 20.66 | 2.91 | 141 | AA | 20.47 | 2.39 | 27 | 0.47 | 0.63 | 0.34 | 0.93 | 0.71 |
| rs2020942 |  | SLC6A4 | GG | 20.53 | 2.35 | 402 | AG | 20.32 | 2.73 | 76 | AA |  |  |  | 0.44 | 0.51 | b |  |  |
| rs8076005 |  | SLC6A4 | AA | 20.46 | 2.30 | 355 | AG | 20.58 | 2.68 | 111 | GG | 20.71 | 3.04 | 12 | 0.16 | 0.85 | 0.63 | 0.72 | 0.86 |
| 5HTTLPR |  | 5HTT | MM | 20.56 | 2.34 | 246 | VM | 20.43 | 2.52 | 187 | VV | 20.34 | 2.43 | 42 | 0.25 | 0.78 | 0.58 | 0.58 | 0.81 |
| rs1497020 |  | SLC18A1(VMAT1) | AA | 20.49 | 2.82 | 133 | AG | 20.47 | 2.21 | 235 | GG | 20.54 | 2.31 | 110 | 0.03 | 0.97 | 0.95 | 0.88 | 0.82 |
| rs1018079 |  | SLC18A1(VMAT1) | AA | 20.29 | 2.26 | 303 | AG | 20.72 | 2.32 | 156 | GG | 22.10 | 4.35 | 18 | 5.92 | < 0.01 | 0.07 | < 0.01 | 0.02 |
| rs12545707 |  | SLC18A1(VMAT1) | GG | 20.51 | 2.44 | 458 | AG | 20.04 | 1.64 | 20 | AA |  |  |  | 0.74 | 0.39 | b |  |  |
| rs903997 |  | SLC18A1(VMAT1) | GG | 20.45 | 2.70 | 142 | CG | 20.51 | 2.30 | 235 | CC | 20.50 | 2.25 | 101 | 0.03 | 0.97 | 0.81 | 0.87 | 0.97 |
| rs2270650 |  | SLC18A1(VMAT1) | GG | 20.28 | 2.27 | 319 | AG | 20.79 | 2.29 | 140 | AA | 21.92 | 4.30 | 19 | 5.77 | < 0.01 | 0.03 | < 0.01 | 0.05 |
| rs3779671 |  | SLC18A1(VMAT1) | GG | 20.40 | 2.42 | 219 | CG | 20.62 | 2.41 | 189 | CC | 20.45 | 2.40 | 70 | 0.42 | 0.66 | 0.37 | 0.89 | 0.62 |
| rs13258461 |  | SLC18A1(VMAT1) | GG | 20.42 | 2.53 | 169 | AG | 20.58 | 2.40 | 215 | AA | 20.43 | 2.23 | 94 | 0.23 | 0.80 | 0.53 | 0.97 | 0.63 |
| rs952858 |  | SLC18A1(VMAT1) | GG | 20.49 | 2.41 | 434 | CG | 20.48 | 2.47 | 44 | CC |  |  |  | < 0.01 | 0.98 | b |  |  |
| rs2270638 |  | SLC18A1(VMAT1) | AA | 20.33 | 2.47 | 344 | AG | 20.92 | 2.20 | 133 | GG |  |  |  | 5.77 | 0.02 | b |  |  |
| rs363338 |  | SLC18A2(VMAT2) | GG | 20.56 | 2.26 | 310 | AG | 20.37 | 2.75 | 154 | AA | 20.40 | 1.64 | 14 | 0.34 | 0.71 | 0.42 | 0.80 | 0.97 |
| rs363222 |  | SLC18A2(VMAT2) | CC | 20.63 | 2.53 | 212 | CG | 20.45 | 2.23 | 210 | GG | 20.21 | 2.61 | 54 | 0.74 | 0.48 | 0.45 | 0.26 | 0.52 |
| rs4752045 |  | SLC18A2(VMAT2) | GG | 20.62 | 2.28 | 148 | CG | 20.37 | 2.46 | 281 | CC | 20.79 | 2.49 | 49 | 0.92 | 0.40 | 0.31 | 0.67 | 0.26 |
| rs363225 |  | SLC18A2(VMAT2) | GG | 20.42 | 2.17 | 147 | AG | 20.59 | 2.55 | 241 | AA | 20.35 | 2.41 | 89 | 0.40 | 0.67 | 0.50 | 0.85 | 0.44 |
| rs363226 |  | SLC18A2(VMAT2) | CC | 20.49 | 2.44 | 393 | CG | 20.51 | 2.27 | 85 | GG |  |  |  | 0.01 | 0.94 | b |  |  |
| rs878567 | Receptor | HTR1A | GG | 20.45 | 2.50 | 316 | AG | 20.56 | 2.25 | 152 | AA | 20.74 | 1.81 | 10 | 0.16 | 0.85 | 0.64 | 0.71 | 0.83 |
| rs10042486 |  | HTR1A | AA | 20.45 | 2.50 | 316 | AG | 20.56 | 2.25 | 152 | GG | 20.74 | 1.81 | 10 | 0.16 | 0.85 | 0.64 | 0.71 | 0.83 |
| rs6297 |  | HTR1B | AA | 20.50 | 2.43 | 387 | AG | 20.46 | 2.33 | 91 | GG |  |  |  | 0.02 | 0.88 | b |  |  |
| rs6298 |  | HTR1B | AA | 20.41 | 2.76 | 131 | AG | 20.57 | 2.24 | 229 | GG | 20.43 | 2.33 | 118 | 0.22 | 0.81 | 0.56 | 0.95 | 0.62 |
| rs2776822 |  | HTR1D | GG | 20.35 | 2.23 | 158 | AG | 20.59 | 2.51 | 242 | AA | 20.46 | 2.47 | 78 | 0.48 | 0.62 | 0.33 | 0.74 | 0.68 |
| rs2746553 |  | HTR1D | AA | 20.35 | 2.23 | 158 | AG | 20.59 | 2.51 | 242 | GG | 20.46 | 2.47 | 78 | 0.48 | 0.62 | 0.33 | 0.74 | 0.68 |
| rs2776815 |  | HTR1D | AA | 20.49 | 2.28 | 268 | AG | 20.51 | 2.65 | 178 | GG | 20.45 | 2.11 | 32 | 0.01 | 0.99 | 0.91 | 0.94 | 0.90 |
| rs6704440 |  | HTR1D | CC | 20.43 | 2.39 | 334 | AC | 20.64 | 2.45 | 131 | AA | 20.56 | 2.55 | 13 | 0.34 | 0.71 | 0.41 | 0.85 | 0.91 |
| rs641032 |  | HTR1D | AA | 20.49 | 2.28 | 268 | AG | 20.51 | 2.65 | 178 | GG | 20.45 | 2.11 | 32 | 0.01 | 0.99 | 0.91 | 0.94 | 0.90 |
| rs604030 |  | HTR1D | AA | 20.49 | 2.28 | 268 | AG | 20.51 | 2.65 | 178 | GG | 20.45 | 2.11 | 32 | 0.01 | 0.99 | 0.91 | 0.94 | 0.90 |
| rs676643 |  | HTR1D | GG | 20.48 | 2.30 | 273 | AG | 20.52 | 2.63 | 173 | AA | 20.45 | 2.11 | 32 | 0.02 | 0.98 | 0.87 | 0.95 | 0.88 |
| rs674386 |  | HTR1D | GG | 20.48 | 2.29 | 269 | AG | 20.53 | 2.65 | 177 | AA | 20.45 | 2.11 | 32 | 0.03 | 0.97 | 0.82 | 0.96 | 0.87 |
| rs2746561 |  | HTR1D | AA | 20.48 | 2.30 | 273 | AG | 20.52 | 2.63 | 173 | GG | 20.45 | 2.11 | 32 | 0.02 | 0.98 | 0.87 | 0.95 | 0.88 |
| rs598438 |  | HTR1D | GG | 20.39 | 2.26 | 165 | AG | 20.57 | 2.47 | 241 | AA | 20.50 | 2.55 | 72 | 0.27 | 0.76 | 0.46 | 0.75 | 0.83 |
| rs1738475 |  | HTR1D | GG | 20.31 | 2.22 | 159 | CG | 20.62 | 2.50 | 242 | CC | 20.46 | 2.49 | 77 | 0.81 | 0.44 | 0.21 | 0.67 | 0.60 |
| rs627304 |  | HTR1D | AA | 20.31 | 2.22 | 159 | AG | 20.62 | 2.50 | 242 | GG | 20.46 | 2.49 | 77 | 0.81 | 0.44 | 0.21 | 0.67 | 0.60 |
| rs2903545 |  | HTR1D | AA | 20.31 | 2.22 | 159 | AG | 20.62 | 2.50 | 242 | GG | 20.46 | 2.49 | 77 | 0.81 | 0.44 | 0.21 | 0.67 | 0.60 |
| rs7630716 |  | HTR1F | CC | 20.57 | 2.25 | 250 | CG | 20.51 | 2.66 | 190 | GG | 19.90 | 2.07 | 38 | 1.30 | 0.27 | 0.78 | 0.11 | 0.15 |
| rs9863076 |  | HTR1F | CC | 20.57 | 2.25 | 250 | AC | 20.51 | 2.66 | 190 | AA | 19.90 | 2.07 | 38 | 1.30 | 0.27 | 0.78 | 0.11 | 0.15 |
| rs1503433 |  | HTR1F | AA | 20.57 | 2.25 | 250 | AC | 20.51 | 2.66 | 190 | CC | 19.90 | 2.07 | 38 | 1.30 | 0.27 | 0.78 | 0.11 | 0.15 |
| rs2016224 |  | HTR1F | AA | 20.57 | 2.25 | 250 | AG | 20.51 | 2.66 | 190 | GG | 19.90 | 2.07 | 38 | 1.30 | 0.27 | 0.78 | 0.11 | 0.15 |
| rs7652406 |  | HTR1F | GG | 20.58 | 2.25 | 251 | AG | 20.50 | 2.67 | 189 | AA | 19.90 | 2.07 | 38 | 1.32 | 0.27 | 0.74 | 0.11 | 0.16 |
| rs7997012 |  | HTR2A | GG | 20.55 | 2.31 | 265 | AG | 20.60 | 2.63 | 173 | AA | 19.68 | 1.95 | 40 | 2.56 | 0.08 | 0.81 | 0.03 | 0.03 |
| rs977003 |  | HTR2A | AA | 20.29 | 2.30 | 299 | AC | 20.92 | 2.62 | 151 | CC | 20.40 | 2.19 | 28 | 3.45 | 0.03 | 0.01 | 0.82 | 0.29 |
| rs6561332 |  | HTR2A | CC | 20.41 | 2.32 | 412 | AC | 21.02 | 2.87 | 66 | AA |  |  |  | 3.64 | 0.06 | b |  |  |
| rs6561333 |  | HTR2A | GG | 20.69 | 2.41 | 150 | AG | 20.46 | 2.46 | 230 | AA | 20.28 | 2.30 | 98 | 0.89 | 0.41 | 0.37 | 0.20 | 0.54 |
| rs9567739 |  | HTR2A | CC | 20.24 | 2.15 | 143 | CG | 20.51 | 2.45 | 218 | GG | 20.77 | 2.61 | 117 | 1.60 | 0.20 | 0.29 | 0.08 | 0.35 |
| rs655888 |  | HTR2A | GG | 20.25 | 2.16 | 150 | AG | 20.53 | 2.45 | 214 | AA | 20.76 | 2.64 | 113 | 1.46 | 0.23 | 0.28 | 0.09 | 0.41 |
| rs7984966 |  | HTR2A | AA | 20.55 | 2.46 | 435 | AG | 19.95 | 1.75 | 43 | GG |  |  |  | 2.40 | 0.12 | b |  |  |
| rs1885884 |  | HTR2A | GG | 20.47 | 2.26 | 266 | CG | 20.59 | 2.71 | 177 | CC | 20.19 | 1.88 | 35 | 0.46 | 0.63 | 0.58 | 0.52 | 0.36 |
| rs9526240 |  | HTR2A | GG | 20.47 | 2.41 | 457 | AG | 20.90 | 2.54 | 21 | AA |  |  |  | 0.62 | 0.43 | 0.43 |  |  |
| rs2224721 |  | HTR2A | CC | 20.12 | 2.12 | 216 | AC | 20.79 | 2.61 | 209 | AA | 20.87 | 2.51 | 53 | 4.85 | 0.01 | < 0.01 | 0.04 | 0.82 |
| rs6561335 |  | HTR2A | AA | 20.47 | 2.40 | 459 | AG | 21.12 | 2.55 | 19 | GG |  |  |  | 1.36 | 0.24 | b |  |  |
| rs1928042 |  | HTR2A | AA | 20.51 | 2.43 | 429 | AC | 20.36 | 2.21 | 49 | CC |  |  |  | 0.16 | 0.69 | b |  |  |
| rs2770293 |  | HTR2A | GG | 20.45 | 2.55 | 295 | AG | 20.50 | 2.11 | 160 | AA | 20.95 | 2.52 | 23 | 0.45 | 0.64 | 0.85 | 0.34 | 0.40 |
| rs9534501 |  | HTR2A | GG | 20.43 | 2.54 | 303 | AG | 20.52 | 2.13 | 153 | AA | 21.14 | 2.40 | 22 | 0.90 | 0.41 | 0.71 | 0.19 | 0.26 |
| rs2770296 |  | HTR2A | AA | 20.46 | 2.60 | 265 | AG | 20.45 | 2.11 | 181 | GG | 21.06 | 2.35 | 32 | 0.95 | 0.39 | 0.96 | 0.18 | 0.18 |
| rs985933 |  | HTR2A | GG | 20.59 | 2.75 | 202 | AG | 20.34 | 2.10 | 209 | AA | 20.74 | 2.19 | 66 | 0.92 | 0.40 | 0.30 | 0.66 | 0.24 |
| rs927544 |  | HTR2A | AA | 20.52 | 2.67 | 236 | AG | 20.39 | 2.10 | 192 | GG | 20.77 | 2.23 | 50 | 0.53 | 0.59 | 0.56 | 0.52 | 0.32 |
| rs1328684 |  | HTR2A | AA | 20.55 | 2.44 | 406 | AG | 20.17 | 2.23 | 72 | GG |  |  |  | 1.56 | 0.21 | b |  |  |
| rs2296973 |  | HTR2A | CC | 20.49 | 2.48 | 212 | AC | 20.46 | 2.47 | 208 | AA | 20.64 | 1.95 | 58 | 0.13 | 0.88 | 0.89 | 0.67 | 0.61 |
| rs2070040 |  | HTR2A | GG | 20.52 | 2.48 | 205 | AG | 20.47 | 2.45 | 210 | AA | 20.55 | 1.98 | 62 | 0.03 | 0.97 | 0.84 | 0.94 | 0.83 |
| rs953451 |  | HTR2A | CC | 20.48 | 2.31 | 237 | CG | 20.64 | 2.48 | 209 | GG | 19.63 | 2.56 | 32 | 2.44 | 0.09 | 0.50 | 0.06 | 0.03 |
| rs17619600 |  | HTR2B | AA | 20.49 | 2.54 | 299 | AG | 20.52 | 2.18 | 165 | GG | 20.33 | 2.31 | 14 | 0.04 | 0.96 | 0.91 | 0.81 | 0.78 |
| rs6437000 |  | HTR2B | AA | 20.30 | 2.13 | 166 | AC | 20.58 | 2.57 | 238 | CC | 20.63 | 2.47 | 74 | 0.81 | 0.45 | 0.25 | 0.33 | 0.88 |
| rs10194776 |  | HTR2B | AA | 20.34 | 2.11 | 174 | AG | 20.58 | 2.59 | 233 | GG | 20.56 | 2.49 | 71 | 0.53 | 0.59 | 0.32 | 0.52 | 0.94 |
| rs16827801 |  | HTR2B | AA | 20.57 | 2.55 | 120 | AG | 20.49 | 2.43 | 243 | GG | 20.41 | 2.22 | 115 | 0.14 | 0.87 | 0.76 | 0.60 | 0.76 |
| rs1549339 |  | HTR2B | AA | 20.29 | 2.11 | 170 | AG | 20.59 | 2.58 | 235 | GG | 20.64 | 2.48 | 73 | 0.92 | 0.40 | 0.22 | 0.31 | 0.88 |
| rs17586428 |  | HTR2B | AA | 20.46 | 2.34 | 288 | AG | 20.53 | 2.52 | 173 | GG | 20.57 | 2.58 | 17 | 0.06 | 0.95 | 0.76 | 0.86 | 0.95 |
| rs2192371 |  | HTR2C | AA | 20.61 | 2.42 | 242 | AG | 19.79 | 1.80 | 124 | GG | 21.03 | 2.78 | 112 | 8.62 | < 0.01 | < 0.01 | 0.12 | < 0.01 |
| rs6644065 |  | HTR2C | AA | 20.45 | 2.26 | 373 | AG | 20.05 | 2.81 | 68 | GG | 21.74 | 2.77 | 37 | 6.30 | < 0.01 | 0.21 | < 0.01 | < 0.01 |
| rs4911871 |  | HTR2C | AA | 20.39 | 2.23 | 350 | AG | 20.17 | 2.74 | 76 | GG | 21.68 | 2.76 | 51 | 7.44 | < 0.01 | 0.47 | < 0.01 | < 0.01 |
| rs2276302 |  | HTR3A | AA | 20.56 | 2.48 | 374 | AG | 20.25 | 2.16 | 103 | GG |  |  |  | 1.30 | 0.26 | b |  |  |
| rs11214769 |  | HTR3B | AA | 20.31 | 2.22 | 335 | AG | 20.88 | 2.85 | 124 | GG | 21.21 | 2.20 | 19 | 3.46 | 0.03 | 0.02 | 0.11 | 0.58 |
| rs1176746 |  | HTR3B | GG | 20.66 | 2.39 | 206 | AG | 20.41 | 2.49 | 225 | AA | 20.12 | 2.08 | 46 | 1.20 | 0.30 | 0.27 | 0.17 | 0.46 |
| rs6808122 |  | HTR3C | AA | 20.37 | 2.49 | 274 | AG | 20.70 | 2.35 | 179 | GG | 20.31 | 1.86 | 25 | 1.09 | 0.34 | 0.15 | 0.90 | 0.44 |
| rs6766410 |  | HTR3C | AA | 20.56 | 2.59 | 177 | AC | 20.50 | 2.38 | 240 | CC | 20.29 | 2.00 | 61 | 0.28 | 0.76 | 0.81 | 0.46 | 0.54 |
| rs6807362 |  | HTR3C | GG | 20.50 | 2.57 | 241 | CG | 20.48 | 2.30 | 204 | CC | 20.53 | 1.93 | 33 | 0.01 | 0.99 | 0.94 | 0.95 | 0.92 |
| rs939334 |  | HTR3D | AA | 20.42 | 2.35 | 247 | AG | 20.52 | 2.51 | 188 | GG | 20.80 | 2.36 | 42 | 0.49 | 0.61 | 0.64 | 0.34 | 0.50 |
| rs10937159 |  | HTR3D | AA | 20.55 | 2.49 | 240 | AC | 20.44 | 2.23 | 183 | CC | 20.42 | 2.65 | 55 | 0.12 | 0.88 | 0.66 | 0.73 | 0.96 |
| rs6792482 |  | HTR3D | GG | 20.63 | 2.34 | 126 | AG | 20.53 | 2.44 | 236 | AA | 20.27 | 2.43 | 116 | 0.75 | 0.47 | 0.70 | 0.24 | 0.33 |
| rs1467257 |  | HTR3D | CC | 20.59 | 2.51 | 290 | AC | 20.38 | 2.30 | 149 | AA | 20.51 | 2.26 | 27 | 0.37 | 0.69 | 0.39 | 0.88 | 0.79 |
| rs7627615 |  | HTR3E | AA | 20.49 | 2.54 | 276 | AG | 20.45 | 2.21 | 177 | GG | 20.75 | 2.30 | 25 | 0.17 | 0.85 | 0.86 | 0.61 | 0.56 |
| rs7432211 |  | HTR3E | AA | 20.42 | 2.55 | 187 | AG | 20.58 | 2.38 | 235 | GG | 20.37 | 2.08 | 56 | 0.31 | 0.73 | 0.50 | 0.88 | 0.55 |
| rs7733401 |  | HTR4 | CC | 20.45 | 2.14 | 123 | AC | 20.56 | 2.55 | 263 | AA | 20.36 | 2.36 | 92 | 0.28 | 0.76 | 0.66 | 0.79 | 0.48 |
| rs3995090 |  | HTR4 | CC | 20.47 | 2.29 | 270 | AC | 20.58 | 2.64 | 174 | AA | 20.26 | 2.13 | 34 | 0.28 | 0.76 | 0.64 | 0.64 | 0.49 |
| rs4597955 |  | HTR4 | AA | 20.51 | 2.41 | 432 | AG | 20.38 | 2.43 | 46 | GG |  |  |  | 0.12 | 0.73 | b |  |  |
| rs10051356 |  | HTR4 | GG | 20.65 | 2.73 | 176 | CG | 20.42 | 2.18 | 232 | CC | 20.34 | 2.28 | 70 | 0.61 | 0.55 | 0.35 | 0.37 | 0.81 |
| rs1883074 |  | HTR4 | GG | 20.46 | 2.37 | 145 | AG | 20.62 | 2.54 | 245 | AA | 20.20 | 2.10 | 88 | 1.02 | 0.36 | 0.53 | 0.42 | 0.16 |
| rs13166761 |  | HTR4 | GG | 20.38 | 2.23 | 244 | AG | 20.79 | 2.64 | 202 | AA | 19.48 | 1.81 | 32 | 4.74 | 0.01 | 0.07 | 0.05 | < 0.01 |
| rs6873382 |  | HTR4 | AA | 20.56 | 2.49 | 151 | AG | 20.55 | 2.45 | 247 | GG | 20.19 | 2.13 | 80 | 0.75 | 0.47 | 0.97 | 0.27 | 0.25 |
| rs7711800 |  | HTR4 | GG | 20.47 | 2.82 | 128 | AG | 20.45 | 2.13 | 241 | AA | 20.60 | 2.49 | 109 | 0.15 | 0.86 | 0.94 | 0.68 | 0.59 |
| rs6580561 |  | HTR4 | AA | 20.71 | 2.44 | 234 | AG | 20.30 | 2.43 | 197 | GG | 20.22 | 2.10 | 47 | 1.88 | 0.15 | 0.08 | 0.20 | 0.82 |
| rs1972644 |  | HTR4 | AA | 20.66 | 2.40 | 165 | AG | 20.49 | 2.56 | 220 | GG | 20.27 | 2.02 | 90 | 0.79 | 0.45 | 0.48 | 0.21 | 0.47 |
| rs1800883 |  | HTR5A | CC | 20.58 | 2.52 | 133 | CG | 20.54 | 2.25 | 219 | GG | 20.31 | 2.58 | 125 | 0.50 | 0.61 | 0.87 | 0.36 | 0.39 |
| rs6320 |  | HTR5A | AA | 20.57 | 2.47 | 183 | AT | 20.50 | 2.47 | 225 | TT | 20.26 | 2.04 | 70 | 0.42 | 0.65 | 0.77 | 0.36 | 0.47 |
| rs732050 |  | HTR5A | AA | 20.44 | 2.28 | 302 | AG | 20.49 | 2.55 | 157 | GG | 21.36 | 3.09 | 19 | 1.29 | 0.28 | 0.84 | 0.11 | 0.14 |
| rs1440449 |  | HTR5A | AA | 20.39 | 2.43 | 191 | AC | 20.45 | 2.27 | 219 | CC | 20.92 | 2.75 | 68 | 1.28 | 0.28 | 0.80 | 0.12 | 0.16 |
| rs11676829 |  | HTR5B | AA | 20.61 | 2.47 | 361 | AG | 20.10 | 2.22 | 107 | GG | 20.36 | 1.58 | 10 | 1.89 | 0.15 | 0.05 | 0.74 | 0.75 |
| rs2245686 |  | HTR5B | GG | 20.49 | 2.45 | 276 | AG | 20.49 | 2.36 | 165 | AA | 20.50 | 2.43 | 36 | < 0.01 | 1.00 | 0.99 | 0.98 | 0.98 |
| rs10917509 |  | HTR6 | AA | 20.59 | 2.51 | 309 | AG | 20.32 | 2.21 | 169 | GG |  |  |  | 1.37 | 0.24 | b |  |  |
| rs4912138 |  | HTR6 | AA | 20.59 | 2.44 | 138 | AG | 20.39 | 2.37 | 216 | GG | 20.57 | 2.45 | 124 | 0.38 | 0.69 | 0.45 | 0.96 | 0.50 |
| rs6658108 |  | HTR6 | GG | 20.46 | 2.24 | 280 | AG | 20.49 | 2.70 | 168 | AA | 20.86 | 2.27 | 30 | 0.37 | 0.69 | 0.91 | 0.39 | 0.44 |
| rs3790757 |  | HTR6 | GG | 20.41 | 2.36 | 348 | AG | 20.72 | 2.54 | 130 | AA |  |  |  | 1.58 | 0.21 | b |  |  |
| rs9659997 |  | HTR6 | GG | 20.56 | 2.52 | 302 | AG | 20.31 | 2.21 | 157 | AA | 20.98 | 2.21 | 19 | 0.97 | 0.38 | 0.29 | 0.46 | 0.25 |
| rs7904569 |  | HTR7 | AA | 20.50 | 2.38 | 206 | AG | 20.69 | 2.53 | 215 | GG | 19.72 | 1.85 | 57 | 3.64 | 0.03 | 0.43 | 0.03 | 0.01 |
| rs4933194 |  | HTR7 | GG | 20.42 | 2.41 | 192 | AG | 20.63 | 2.44 | 227 | AA | 20.23 | 2.29 | 59 | 0.81 | 0.45 | 0.38 | 0.59 | 0.26 |
| rs11596518 |  | HTR7 | AA | 20.52 | 2.56 | 304 | AG | 20.48 | 2.07 | 154 | GG | 20.24 | 2.64 | 20 | 0.13 | 0.88 | 0.87 | 0.62 | 0.68 |
| rs1573935 |  | HTR7 | AA | 20.36 | 2.36 | 132 | AG | 20.64 | 2.49 | 241 | GG | 20.33 | 2.29 | 105 | 0.92 | 0.40 | 0.27 | 0.93 | 0.27 |
| rs10881838 |  | HTR7 | AA | 20.36 | 2.16 | 164 | AG | 20.53 | 2.48 | 230 | GG | 20.66 | 2.68 | 84 | 0.47 | 0.62 | 0.50 | 0.36 | 0.67 |
| rs12249377 |  | HTR7 | CC | 20.43 | 2.36 | 344 | AC | 20.64 | 2.50 | 124 | AA | 20.98 | 3.09 | 10 | 0.56 | 0.57 | 0.40 | 0.48 | 0.67 |
| rs10785973 |  | HTR7 | CC | 20.49 | 2.40 | 240 | AC | 20.59 | 2.49 | 201 | AA | 19.96 | 2.01 | 37 | 1.09 | 0.34 | 0.66 | 0.21 | 0.14 |

Note: Empty cells mean no such genotypes were found in our sample. Maj: Major allele; Het: Heterozygote; Min: Minor allele.

aResults of post hoc comparisons. mh = Maj versus Het, mm = Maj versus Min, hm= Het versus Min.

bPost hoc comparison was not run because there were only 2 groups for this locus.
